# Diagnostic value and safety of contact laser-assisted endobronchial ultrasound-guided tunnel drilling biopsy in mediastinal and hilar lymphadenopathy: a retrospective study

**DOI:** 10.3389/fmed.2025.1520690

**Published:** 2025-01-30

**Authors:** Wenyu Zhan, Tian Wang, Changqing Yang, Yubao Wang, Nansheng Wan, Jing Feng

**Affiliations:** Respiratory Department, Tianjin Medical University General Hospital, Tianjin, China

**Keywords:** mediastinal lymphadenopathy, endobronchial ultrasound-guided tunnel drilling biopsy, contact laser-assisted biopsy, diagnostic yield, lymph node pathology

## Abstract

**Introduction:**

Mediastinal and hilar lymphadenopathies are primarily diagnosed pathologically. Contact laser-assisted endobronchial ultrasound-guided tunnel drilling biopsy (EBUS-TDB), which uses a laser as a tunneling and incision tool, may yield more satisfactory specimens than conventional endobronchial ultrasound-guided transbronchial needle aspiration (EBUS-TBNA), thereby improving the diagnostic yield. This study aims to evaluate the diagnostic value and safety of contact laser-assisted EBUS-TDB compared to EBUS-TBNA in the assessment of mediastinal and hilar lymph nodes.

**Methods:**

This retrospective study included patients who presented to our hospital between October 2022 and April 2024 with mediastinal or hilar lymph nodes of short diameter ≥ 1 cm on computed tomography (CT) or abnormally increased lymph node metabolism on positron emission tomography (PET)-CT. All patients underwent both EBUS-TBNA and EBUS-TDB procedures successively.

**Results:**

Overall, 278 patients were included in the study, and 244 cases were confirmed. The diagnostic rates (*p*-values) for EBUS-TDB and EBUS-TBNA in pulmonary and extrapulmonary malignancies, lymphoma, sarcoidosis, and lymph node tuberculosis were 96.6% vs. 76.3% (0.043), 100% vs. 67.7% (−), 88.9% vs. 31.1% (0.555), and 69.2% vs. 30.8% (0.049), respectively. No serious adverse events occurred during or after either procedure.

**Conclusion:**

Contact laser-assisted EBUS-TBNB demonstrates superior diagnostic performance compared to EBUS-TBNA for the evaluation of mediastinal or hilar lymph nodes, making it an alternative to EBUS-TBNA for enhanced diagnostic precision.

## 1 Introduction

**Figure 1:**
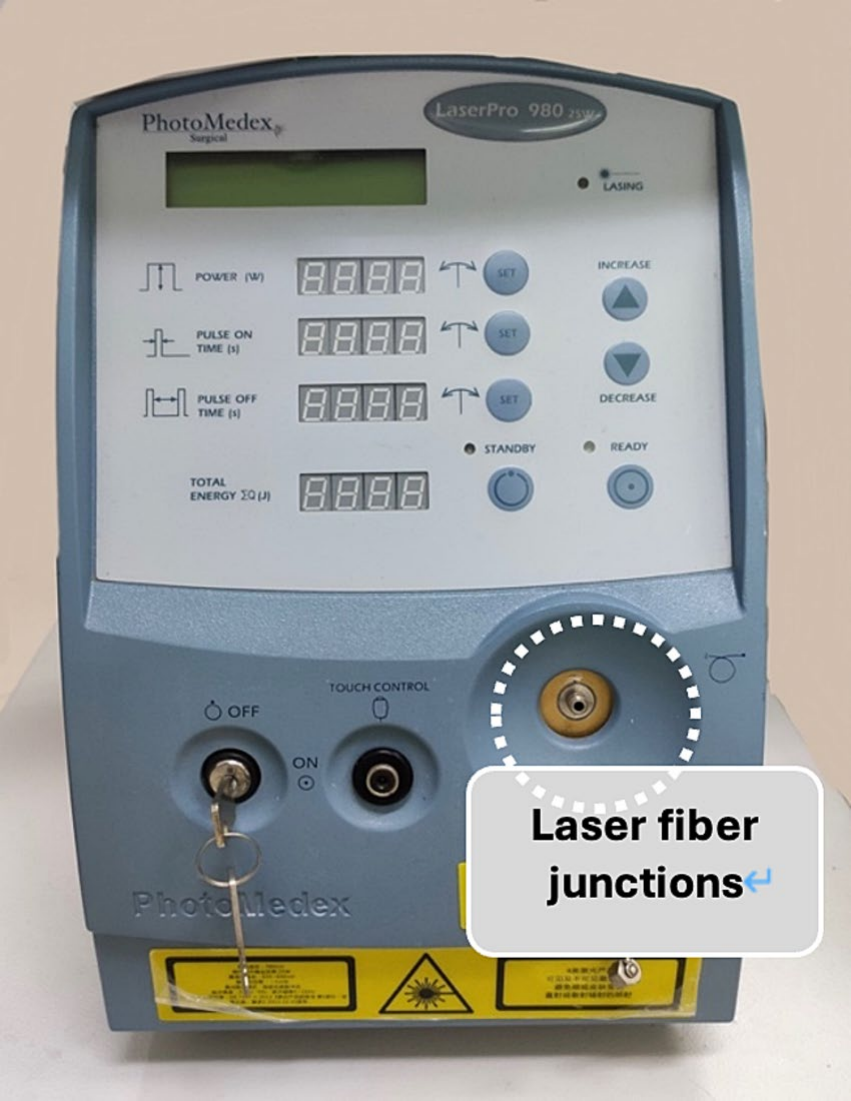
Contact laser fine optical fibers (CFE0.6-SMA).

Various benign and malignant lesions cause mediastinal and hilar lymphadenopathy, with malignant tumors and granulomas being the most common causes (1). Endobronchial ultrasound-guided transbronchial needle aspiration (EBUS-TBNA) is the preferred minimally invasive technique for diagnosing mediastinal lesions. EBUS-TBNA offers good diagnostic yield and operational safety; however, it has an inherent limitation: the number of tissue samples obtained by fine needle aspiration is limited, making it suitable primarily for cytological rather than histopathological diagnosis. Previous studies have reported a diagnostic yield of EBUS-TBNA for benign lesions and lymphomas ranging from 54 to 93% (2–5). New techniques for obtaining lymph node tissues include EBUS-guided intranodal forceps biopsy (EBUS-IFB) and EBUS-guided cautery-assisted transbronchial forceps biopsy (ca-TBFB), which are complementary techniques (6). These techniques have been shown to improve the diagnostic yield of sarcoidosis and lymphoma (3, 7). Combining EBUS-IFB with EBUS-TBNA has been demonstrated to improve the diagnostic yield for benign mediastinal lymphadenopathy (8). However, EBUS-IFB relies on the tract created by EBUS-TBNA to access lymph nodes, which limits the size of the biopsy forceps and affects the number of biopsy specimens obtained, necessitating an increased number of biopsies (5, 9). ca-TBFB addresses the limitations of EBUS-IFB by using cautery. However, prospective studies have shown that ca-TBFB has lower sensitivity for detecting malignant tumors compared to EBUS-TBNA (10). At our center, we improved the tunnel drilling procedure by performing ultrasound bronchoscopy-guided mediastinotomy biopsy using contact laser fine optical fibers (CFE0.6-SMA) as tunneling and incision tools (Figure 1). Repeated biopsy sampling using standard forceps biopsy was performed four to six times to obtain satisfactory tissue samples. This study aimed to evaluate the diagnostic value and safety of CFE0.6-SMA-assisted endobronchial ultrasound-guided tunnel drilling biopsy (EBUS-TDB) compared to EBUS-TBNA for mediastinal and hilar lymph nodes.

## 2 Methods

### 2.1 Study design

We conducted a retrospective cohort analysis and included patients referred to Tianjin Medical University General Hospital for bronchoscopy between October 2022 and April 2024 who met the following criteria: (1) age ≥ 18 years; (2) computed tomography (CT) showing mediastinal or hilar lymph nodes with a short diameter ≥ 1 cm, or positron emission tomography (PET)-CT revealing abnormally increased lymph node metabolism; and (3) completion of both EBUS-TBNA and EBUS-TDB procedures in the same patient. The exclusion criteria were as follows: (1) heart, brain, kidney, lung, and other important organ dysfunction; (2) coagulopathy and severe blood system diseases; and (3) active bleeding. This study was approved by the Ethics Review Board of Tianjin Medical University General Hospital (IRB2023-YX-265-01). Informed consent was obtained from all patients or their legal guardians prior to surgery. This study was conducted in accordance with the guidelines outlined in the Declaration of Helsinki.

### 2.2 Surgical method

Preoperatively, the target lymph nodes were selected based on imaging findings, and patients were assessed for suitability for anesthesia. Following adequate general anesthesia using propofol (1.5–2.5 mg/kg, Corden Pharma S.P.A, Ireland) and muscle relaxation with rocuronium bromide injection (0.6 mg/Kg, Guangdong Jiabo Pharmaceutical Co., Ltd., China), a rigid bronchoscope or laryngeal mask airway was placed. Oxygen inhalation or high-frequency ventilation was given, and the vital signs of the patient were monitored using an electrocardiogram. Initially, we observed the airways using conventional bronchoscopy (BF-260; Olympus, Tokyo, Japan). Some patients underwent mucosal or lung biopsy and bronchoalveolar lavage. This was followed by ultrasound bronchoscopy (CP-EBUS; BF-UC26 0F-OL8; Olympus, Japan). The surgeon used ultrasound images to identify the target area, avoiding blood vessels and necrotic areas and selecting soft and elastic parts of the surgical area, such as the tracheal cartilage ring, whenever possible.

Standard Olympus EBUS TBNA 22G puncture needle (NA – 201SX – 4022; Olympus, Japan) was used to perform EBUS-TBNA three to four times consecutively, with each puncture lasting approximately 3 min and a total duration of 10–15 min to ensure consistent puncture sampling position. The specimen was sent to the pathology department after forming a liquid wax block in the cell preservation solution.

**Figure 2:**
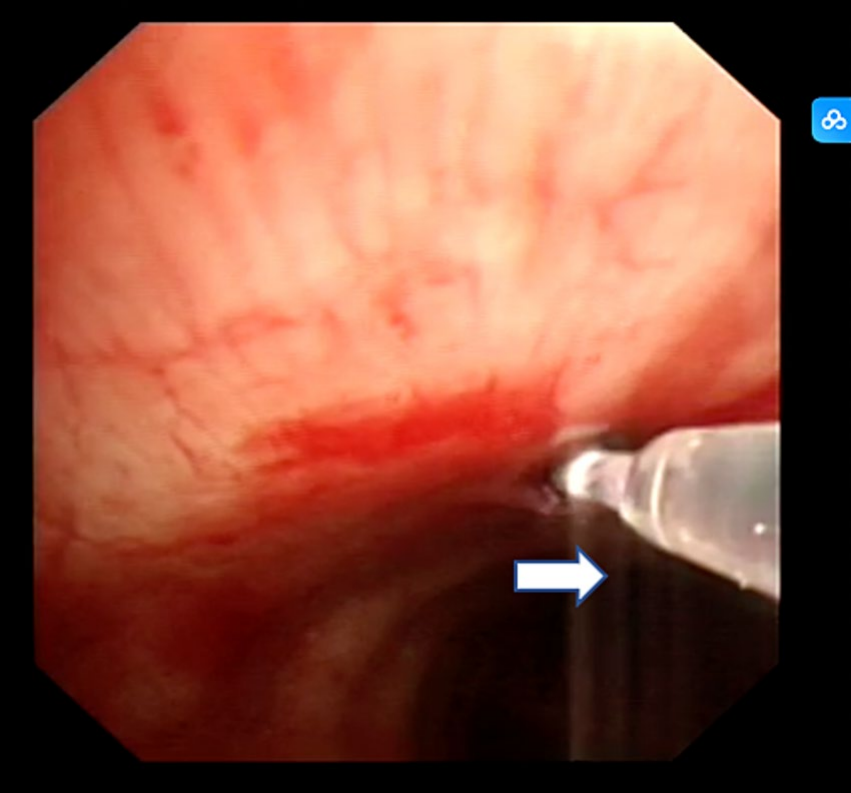
Contact laser tunnel drilling biopsy. The white arrow represents contact laser fibers.

**Figure 3:**
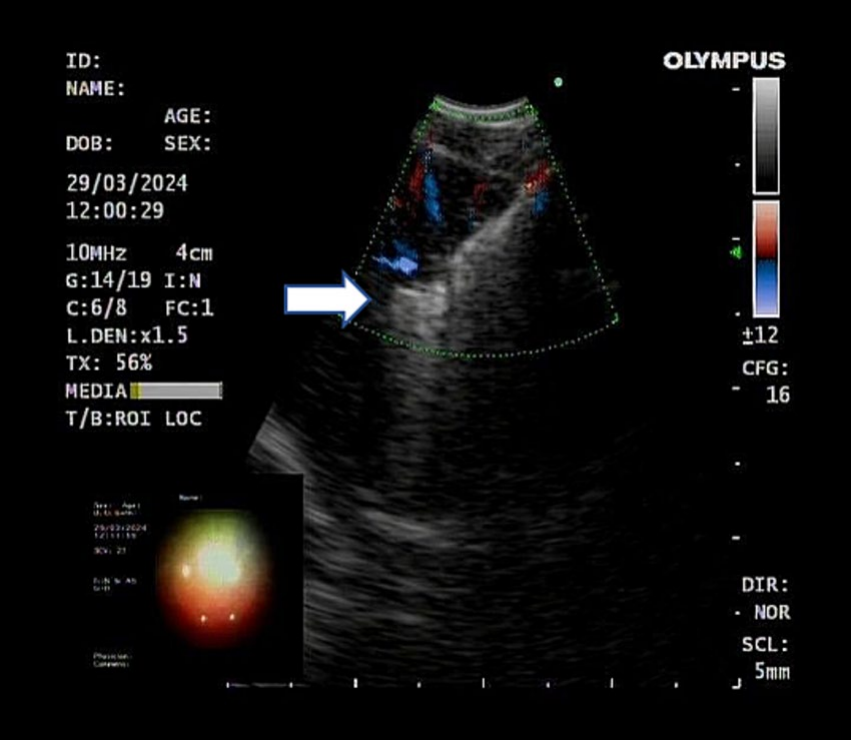
Ultrasound showed the biopsy forceps passing through the tunnel into the lymph nodes. The white arrow represents the open biopsy forceps.

After completing EBUS-TBNA, EBUS-TDB was performed using a Maidishi contact laser CFE0.6-SMA fine fiber for tunneling and incision. A thin laser fiber was preinstalled in the EBUS endoscope and positioned slightly ahead of the treatment orifice, and the output power was adjusted to 7.5 W. A thin fiber was inserted through the previous EBUS-TBNA puncture imprint, and the fiber tip dissected the mucosal tissue in layers, creating a meticulous full-thickness incision (Figure 2). This tunnel allowed the passage of the 1.8 mm standard biopsy forceps (RXQY-W1216-PA, TIANJINGUANGYUANFUTIAN, China), with laser tunneling taking approximately 5 min. The standard biopsy forceps were preloaded into the EBUS endoscope and inserted into the tunnel inlet. The forceps were then passed through the tunnel inlet to penetrate deep into the target area (Figure 3). Samples were taken four to six times, with each biopsy lasting approximately 3 min, for a total duration of 8–10 min. Tissues were preserved in formalin and sent to the pathology department.

Intraoperative bleeding was usually controlled by bronchoscopic compression, ice saline, or thrombin (Changchunlei Yunshang Pharmaceutical Co., Ltd., 2000 units). At the end of the procedure, the surgical area was inspected for complications, such as significant bleeding. The rigid bronchoscope was removed, and a laryngeal mask airway was placed and connected to an anesthesia device for ventilation. The patient was allowed to recover and was monitored in the bronchoscopy resuscitation room for at least 2 h.

### 2.3 Postoperative adverse events

Patients were monitored for hemoptysis, fever, hypoxia, arrhythmia, chest pain, dyspnea, and other symptoms within 24 h post-surgery. Body temperature was tracked for 1 week post-surgery, and a chest CT was performed when necessary.

### 2.4 Pathological diagnosis

The liquid wax blocks and tissue specimens were sent to the pathology department for dehydration, transparency, wax immersion, sectioning, staining, and sealing. Immunohistochemistry was performed according to clinical classification. Pathological diagnoses were made by an attending physician and reviewed by a consultant.

### 2.5 Criteria for diagnosis

Benign and malignant tumors and lymphomas were diagnosed based on pathological findings. Tuberculosis was diagnosed using pathological findings or positive results from etiological examinations, including PCR, GeneXpert, or mNGS tests. Sarcoidosis diagnosis was based on three major criteria: a compatible clinical presentation, evidence of non-necrotizing granulomatous inflammation in one or more tissue samples, and exclusion of alternative causes of granulomatous diseases (11).

### 2.6 Statistical analysis

Data analysis was conducted using IBM SPSS Statistics for Windows, version 26.0 (IBM Corp., Armonk, N.Y., United States). Normally distributed continuous variables are presented as means and standard deviations, with *t*-tests used for group comparisons. Skewed continuous variables, medians, and interquartile ranges were reported, and Wilcoxon rank tests were used. Categorical variables are presented as frequencies and percentages, with chi-square tests used to analyze the differences between the two groups of data. A *p*-value of <0.05 was considered significant.

## 3 Results

### 3.1 Patient characterization

Overall, 278 patients underwent both EBUS-TBNA and laser transbronchial node biopsy (TBNB) examinations, including 244 confirmed cases (positive results from pathology of the lung, mucosa, lymph node biopsy, or needle aspiration). A total of 149 men and 95 women, with a mean age of 65 years (range: 21–83) and a mean BMI of 24.18 ± 3.78 kg/m^2^ were included. Among these patients, 91 had a smoking index >400 years, and the mean short axis of the lymph node was 1.77 ± 0.55 cm. Patients had the following underlying diseases: 33 with type 2 diabetes, 25 with COPD, 25 had coronary heart disease, and 51 had hypertension. Alveolar lavage was performed in 89 patients, while lung or mucosal biopsies were conducted in 164 patients.

### 3.2 Location of lymph nodes aspirated or biopsied

A total of 326 operating areas were identified among the 278 patients, with the subcarinal lymph node area (zone 7) being the most frequently accessed location (Table 1).

**Table 1:** Location of lymph nodes aspirated or biopsied.

Location	2R	3p	4R	4L	7	10R	10L	11R	11L
Quantity	2	41	9	1	176	2	12	29	27

### 3.3 Diagnostic yield

Among the 244 confirmed cases, diagnoses included 177 pulmonary and extrapulmonary malignant tumors, six lymphomas, 45 cases of sarcoidosis, 13 tuberculosis cases, one mediastinal schwannoma, one lymphoproliferative disease, and one fibrotic lesion. Diagnostic rates are shown in Table 2.

**Table 2:** Diagnostic yield of endobronchial ultrasound-guided tunnel drilling biopsy (EBUS-TDB) and endobronchial ultrasound-guided transbronchial needle aspiration (EBUS-TBNA) in different mediastinal and hilar lymph node lesions.

	Diagnostic rate for EBUS-TDB (%)	Diagnostic rate for EBUS-TBNA (%)	*p*-value
Pulmonary and extrapulmonary malignant tumors	96.6 (171/177)	76.3 (135/177)	0.043
Lymphomas	100 (6/6)	67.7 (4/6)	-
Sarcoidosis	88.9 (40/45)	31.1 (14/45)	0.555
Tuberculosis	96.2 (9/13)	30.8 (4/13)	0.049

Overall, 177 cases of pulmonary and extrapulmonary malignant tumors were diagnosed, and the diagnostic rates of EBUS-TDB and EBUS-TBNA were 96.6% (171/177) and 76.3% (135/177), respectively (*p* = 0.043). The final diagnoses were as follows: 46 cases of small cell carcinoma, 71 cases of adenocarcinoma, 33 cases of squamous cell carcinoma, one case each of adenosquamous carcinoma, sarcomatoid carcinoma, acinar cell carcinoma, and undifferentiated carcinoma, eight cases of poorly differentiated carcinoma, nine cases of non-small cell carcinoma (unclassified), and four cases of extrapulmonary metastatic carcinoma. Lymphoma was diagnosed in six cases, and the diagnostic rates of EBUS-TDB and EBUS-TBNA were 100 and 67.7%, respectively. Sarcoidosis was diagnosed in 45 cases, with diagnostic rates of 88.9% for EBUS-TDB and 31.1% for EBUS-TBNA (*p* = 0.555). Lymph node tuberculosis was diagnosed in 13 cases, with diagnostic rates of 69.2% for EBUS-TDB and 30.8% for EBUS-TBNA (*p* = 0.049).

### 3.4 Adverse event

During the procedure, seven patients required hemostasis with ice-following lymph node biopsy, and the other patients had no complications. Post-surgery, 23 patients experienced minor bleeding that did not necessitate hemostatic control; two patients had decreased blood oxygen, which improved with oxygen therapy; one patient developed atrial fibrillation; two patients experienced nausea and vomiting, which resolved with medication; three patients developed postoperative low-grade fever, which subsided spontaneously; and one patient experienced mandibular joint insufficiency, which could be reduced by manipulation. No cases of fever were observed 1 week post-surgery, and no mediastinal infection was observed based on clinical symptoms.

## 4 Discussion

Our findings showed that the diagnostic yield of EBUS-TDB for malignancies and tuberculosis in the mediastinal and hilar lymph nodes was significantly higher than that of EBUS-TBNA. While the EBUS-TDB group also showed significantly higher diagnostic yield for sarcoidosis and lymphoma compared to the EBUS-TBNA group, the results did not meet the statistical criteria. The overall serious adverse effects were low, and safety was high in all patients who underwent both procedures.

The diagnostic rate of EBUS-TDB for malignant tumors was higher. First, we speculated that EBUS-TDB allows standard forceps to clamp the lymph node tissue to obtain larger tissue samples by tunneling biopsies compared to conventional microforceps (12). Second, we chose the subcarinal and hilar lymph node areas for the operation; compared with the paratracheal position, the puncture angle was smaller and easier to operate. Prospective studies have shown that EBUS-TBNA has a higher diagnostic yield than ca-TBFB and TBFB in confirming the diagnosis of malignancy (100% vs. 78%), particularly non-small cell lung cancer (100% vs. 73%), which is inconsistent with our findings (5, 10). On the contrary, a recently published meta-analysis has shown the superiority of TBNB by forceps or cryoprobes over EBUS-TBNA (13). This superiority may be ascribed to our use of the 22G needle in EBUS-TBNA. Studies have shown that the 21G needle yields a significantly greater number of tumor cells than those obtained with the 22G needle, and better preservation of tissue structure has been observed in patients with malignancies using the 21G needle (14).

The diagnostic yield of EBUS-TBNA for intrathoracic tuberculous lymphadenitis (TBLA) varies widely between centers, ranging from 33 to 83% (15–17). TBLA is usually isolated from mediastinal lymph node involvement, lacks typical clinical and imaging features, and has a low yield of microbial culture alone or pathology, which requires a combination of Xpert or PCR results. Definitive diagnosis is essential to exclude sarcoidosis or malignancies such as lymphoma and lung cancer (18, 19). Our study improved the diagnostic yield for TBLA using EBUS-TDB by obtaining complete tissue samples with characteristic caseating granuloma signs, avoiding the need for invasive surgical procedures.

EBUS-IFB and ca-TBFB combined with EBUS-TBNA have been demonstrated to perform better in the diagnosis of sarcoidosis (7, 20, 21). However, studies comparing the value of EBUS-TDB and EBUS-TBNA alone in the diagnosis of sarcoidosis are lacking. The diagnostic yield of EBUS-TDB for sarcoidosis was higher than that of EBNS-TBNA, and the clinical difference was significant; however, the results did not meet the statistical criteria, which was considered to be related to the small sample size and large data variation. The low diagnostic rate of EBUS-TBNA was due to the pathological findings in sarcoidosis, which consists of non-necrotizing granulomatous tissue. EBUS-TBNA punctures lymph node cells, resulting in incomplete tissue blocks and a lack of typical pathological changes. Additionally, when the tissue block and cell wax were obtained simultaneously from the same patient, the pathology department may prioritize examining larger tissues. The diagnostic yield of EBUS-TDB for lymphoma was 100%, which was higher than that of EBUS-TBNA. However, statistical comparison was limited due to the small sample size (six cases).

The surgeon used the contact laser fine optical fiber as the tunneling tool which allowed energy release only at the optical fiber tip. This tip had to directly contact the mucosal tissue to produce the cutting effect. Consequently, the cutting range was finer, the surgical area was small, and safety was high. The laser created a straighter tunnel, facilitating the passage of standard biopsy forceps through the clamped tissue and yielding more complete tissue samples. This improvement enhanced the diagnostic rate of benign and malignant mediastinal and hilar lymph node lesions while avoiding repeated biopsies and unnecessary invasive thoracoscopic surgery.

Electrosurgical tunnel construction is commonly performed for this purpose, but our center alternates between the two methods. According to the surgeon’s experience, electrosurgical tunnel construction can easily lead to coking, which affects subsequent imaging detection. Conversely, laser cutting involves gasification and results in less coking. However, laser tunnel construction is more precise than that with an electrosurgical knife, and it demands high skill levels from surgeons. Currently, comparative studies between these two methods are limited.

Furthermore, emerging tunneling and sampling methods, including cryo-biopsy and one-time puncture dilatation catheter tunneling, require further comparative study to determine whether they can completely replace traditional EBUS-TBNA or whether they can improve the diagnostic rate compared with laser tunneling (22, 23). In this study, the mean short axis of the lymph node was 1.77 ± 0.55 cm, which was smaller than those observed in cryobiopsy studies (23). However, cryo-biopsy, which employs a 1.1-mm flexible cryoprobe, smaller than standard biopsy forceps, is theoretically applicable to smaller lymph nodes. Similarly, when manipulation of vascular lymph nodes was necessary, bleeding was stopped by balloon compression, whether through clamping or freezing methods.

Neither mediastinoscopy nor postoperative pathology was used as the gold standard in this study. However, the purpose of our study was to compare the differences in diagnostic yield between the two biopsy methods. Apart from tuberculosis cases, all cases were confirmed through lung, mucosal, and lymph node biopsies or pathological puncture results. Thirty-four patients with unclear pathological and clinical follow-up diagnoses were excluded from the study. Consequently, the comparative diagnostic value in our study was limited to patients with a definite diagnosis, precluding a sensitivity and specificity comparison between EBUS-TDB and EBUS-TBNA.

This study has some limitations. This was a retrospective study that lacked standardization of the pathological procedures. For example, pathology departments tended to prioritize examining larger tissue samples, resulting in a low diagnostic rate of EBUS-TBNA. Additionally, the follow-up data of some patients were insufficient, hindering the verification of diagnostic accuracy. In this study, different surgeries were performed on the same patient, and some patients routinely underwent alveolar lavage or mucosal and lung biopsies before lymph node puncture and biopsy. This may have affected the subsequent evaluation of postoperative adverse events. Due to the lack of routine postoperative imaging data, we could only assess whether the patient has a mediastinal infection, emphysema, or other conditions based on clinical symptoms.

## 5 Conclusion

Contact laser-assisted EBUS-TBNB demonstrated superior diagnostic accuracy compared to EBUS-TBNA for evaluating mediastinal or hilar lymph nodes and may be used as an alternative to EBUS-TBNA.

## Data Availability

The datasets generated and/or analyzed during the current study are not publicly available due to privacy concerns related to patient data. Access to these data can be provided upon reasonable request to the corresponding author, subject to ethical approval and data protection regulations.
